# SSRP1 influences colorectal cancer cell growth and apoptosis via the AKT pathway

**DOI:** 10.7150/ijms.38439

**Published:** 2019-10-21

**Authors:** Qian Wang, Shengnan Jia, Yan Jiao, Libo Xu, Ding Wang, Xuyang Chen, Xindan Hu, Hang Liang, Naiyan Wen, Shengnan Zhang, Baofeng Guo, Ling Zhang

**Affiliations:** 1 Department of Pathophysiology, College of Basic Medical Science, Jilin University, Changchun 130021, P. R. China;; 2 Department of Hepatopancreatobiliary Medicine, The Second Hospital of Jilin University, Changchun 130041, P. R. China;; 3 Department of Hepatobiliary and pancreatic surgery, First Hospital of Jilin University, Changchun 130021, P. R. China;; 4 Department of Plastic Surgery, China-Japan Union Hospital, Jilin University, Changchun 130033, P. R.China.

**Keywords:** SSRP1, proliferation, apoptosis, AKT pathway

## Abstract

Colorectal cancer is one of the most common cancers worldwide with a high incidence rate. Therefore, the molecular basis of colorectal tumorigenesis and evolution must be clarified. Structure-specific recognition protein 1 (SSRP1) is involved in transcriptional regulation, DNA damage repair, and cell cycle regulation and has been confirmed to be highly expressed in various tumor tissues, including colorectal cancer. However, the role of SSRP1 in the development of colorectal cancer remains unclear. Therefore, this study explored the role of SSRP1 in the occurrence and development of colorectal cancer. Using bioinformatics databases, including samples from the Cancer Genome Atlas (TCGA), we confirmed high *SSRP1* expression in human colorectal adenocarcinoma tissues. We demonstrated that *SSRP1* knockdown via small interfering RNA significantly inhibited the proliferation of colorectal cancer cells and promoted apoptosis through the AKT signaling pathway, suppressing the invasion and migration of colorectal cancer cells* in vitro* and* in vivo*. In conclusion, this study demonstrated that *SSRP1* silencing influenced the proliferation and apoptosis of colorectal cancer cells via the AKT signaling pathway.

## Introduction

Colorectal cancer is one of the most common malignant tumors of the digestive system and is the second-ranked tumor for cancer deaths globally, with an increase of more than 1 million new cases annually 1, 2. Radiotherapy, chemotherapy, molecular-targeted therapy, and palliative surgery are current treatment options for patients with colorectal tumor metastases; however, the 5-year survival rate remains less than 10%^3^,^4^. Therefore, further research on the pathogenesis of colorectal cancer is of great significance for preventing its occurrence and improving prognosis.

Structural-specific recognition protein 1 (SSRP1) is involved in transcriptional regulation, DNA damage repair, and cell cycle regulation 5. SSRP1 is highly expressed in a variety of tumor tissues, while it is lowly expressed in normal mature tissues 6. Curaxins, compounds with activity against SSRP1, have been shown to induce apoptosis in tumor cells 7. Downregulation of *SSRP1* by siRNA technology can also inhibit the proliferation of U87 and U251 glioma cells through the MAPK pathway 8. SSRP1 is suggested to play a role in the occurrence and development of tumors and thus provides a large platform for further studying the mechanisms of colorectal cancer development.

In human colorectal cancer, whether SSRP1 plays a critic role and its underlying mechanisms of tumor genesis and evolution is unclear. To clarify it, we first analyze the SSRP1 expression by TCGA databases and cell lines, and identify its influence on cell proliferation and apoptosis by AKT pathway. Besides, *in vitro* and *in vivo* experiments also prove its migration and invasion influence. Therefore, SSRP1 may providesa possible therapeutic strategy and diagnostic targets on human colorectal cancer in clinic.

## Materials and methods

### Bioinformatics

GEPIA (*http://gepia.cancer-pku.cn/index.html*) and UALCAN browser (*http://ualcan.path.uab.edu/index.html*), developed interactive web servers, were used for analyzing data of samples from the TCGA.

### Cell culture

Human colorectal cancer cell lines including DLD1, SW620, HCT15, HCT116, HCT8 and a normal human colon mucosal epithelial cell line NCM460 were gifted by the School of Public Health, Jilin University (Jilin, China). The cell lines were all cultured in RPMI 1640 medium (Gibco, Thermo Fisher Scientifc, Inc., Waltham, MA, USA), supplemented with 10% foetal bovine serum (FBS, BI, Israel), 100 U/mL penicillin and 100 mg/mL streptomycin (Sigma, St. Louis, MO, USA). Cells were cultured at 37°C in a humidified atmosphere containing 5% CO_2_.

### Transient transfection

SW620 and HCT15 cells were transfected with siRNAs, which were purchased from Genepharma (Suzhou, China) and the sequences of siRNAs were as follows: siSSRP1-1 sense, 5'-GCCAUGUCUACAAGUAUGATT-3' and antisense, 5'-UCAUACUUGUAGACAUGGCTT-3'; siSSRP1-2 sense, 5'-CCCAGAAUGGUGUUGUCAAATT-3' and antisense, 5'-UUUGACAACACAUUCUGGGTT-3'; negative control sense, 5'‑CACGCAGAACGTGAACACC‑3' and antisense, 5'‑GGCAGTAGATAACGTGAGGGA‑3'. Prior to transfection, cells were cultured in 96-well plates or 6-well plates ensuring that these cells had reached 80% confluency the next day. The thermo transfection agent Interferin (Thermo Fisher Scientific, Inc., Waltham, MA, USA) was used according to the manufacturer's protocol. Cells were collected after transfection at 48-72 h.

### Cell viability, proliferation and colony formation assay

SW620 and HCT15 cells were plated onto 96-well plates at a concentration of 1×10^4^ cells/well to detect cell viability using Cell Counting Kit-8 (CCK-8; MCE, New Jersey, USA) at 24 h, 48 h and 72 h after transfection. The OD450 was measured by FLUO star Omega reader (BMG LABTECH, Ortenberg, Germany). Cells were seeded on 6-well plates after transfection at a concentration of 5×10^4^ cells/well and cell growth was assessed by growth counting at 24 h, 48 h and 72 h. For colony formation assay, 100 cells/well were plated on 6-well plates and in 5-7 days after transfection the colonies were fixed with 4% paraformaldehyde and then stained with 0.2% crystal violet. The number of colony formation was measured by IX71 inverted fluorescence microscope (Olympus, Shinjuku, Tokyo, Japan).

### Cell cycle analysis

Cells were collected by trypsinization and washed twice with cold PBS. After fixation in ice-cold 75% ethanol for one hour at -20℃ or overnight at 4℃, cells were washed with cold PBS and resuspended with 500 µL cold PBS. Then 20 µL RNase A solution was added and incubated in a 37 ℃ water bath for 30 minutes. Each sample was stained with 400 µL PI stain solution (BestBio, ShangHai, China) at 4℃ for 60 min in the dark. Cell cycle distributions were detected by Accuri C6 flow cytometer (Becton Dickinson, Franklin Lakes, New Jersey, USA).

### Apoptosis analysis

SW620 and HCT15 cells were plated onto 6-well plates at a concentration of 1×10^5^ cells/well. Transfection experiments were performed on the following day. SH-6 (10uM), an inhibitor of AKT, was obtained from Abcam. Cells were harvested in 48 h and washed twice with cold PBS. Subsequently cells were resuspended and stained with 5 µL Annexin-V-FITC and 5 µL PI (50 μg/mL) of Annexin V FITC Apop Dtec Kit (BD Biosciences, San Jose, CA, USA) in the dark at room temperature for 15 min and detected by Accuri C6 flow cytometer (Becton Dickinson, Franklin Lakes, New Jersey, USA).

### Cell migration and invasion assays

Cell migration assay was conducted using Transwell chambers (Coastor, Corning, USA) which were 8-mm pore size. The lower chamber was filled with RPMI 1640 medium containing 20% FBS (BI, Israel). However, the upper chamber was plated cells resuspended in serum-free DMEM and the concentration of every well was 5×10^4^ cells. After cultivation in 5% CO_2_ at 37℃ for 48 hours, the bottom surface of the polycarbonate membranes in the upper chamber were wiped with cotton swabs to remove residual cells and cells were counted visually using IX71 inverted fluorescence microscope (Olympus, Shinjuku, Tokyo, Japan) after stained with 0.1% crystal violet dye. In contrast, matrigel (BD Biosciences, San Jose, CA, USA) was used in the transwell chambers (Coastor, Corning, USA) before cells were plated in the invasion assay. Cell migration and invasion were determined by counting five random fields under an optical microscope and the data are presented as mean ± standard deviation (SD).

### Western blot analysis

Cells and tissues were harvested and lysed in RIPA buffer for 30 min at 4-8ºC. Proteins were detected and quantified using BCA protein assay kit (Thermo Fisher Scientific). Protein samples (30-50 μg) were separated by 12% SDS-PAGE and transferred onto polyvinylidene fluoride membranes. Nonspecific binding sites were blocked with 5% low-fat milk and 0.1% Tween-20 at room temperature for 2 h. Subsequently, the membranes were incubated overnight at 4°C with the following primary antibodies: Proteintech (Wuhan, China): anti-SSRP1 (1:1000; 15696-1-AP), anti-p27 (1:1000; 25614-1-AP), anti-p21 (1:1000; 10355-1-AP), anti-CyclinD1 (1:1000; 60186-1-Ig), anti-BCL2 (1:1000; 26593-1-AP), anti-BAX (1:1000; 50599-2-Ig), anti-MDM2 (1:1000; 19058-1-AP), anti-p53 (1:1000; 10442-1-AP), anti-AKT (1:1000; 60203-2-Ig), anti-β-actin (1:2000; 66009-1-Ig) and arigo (Taiwan, China): anti-P-AKT (1:1000; ARG51559); secondary antibody: goat anti-Mouse (1:2000; 10828-I-AP) and goat anti-Rabbit IgG (H+L) (1:2000; SA0000I-2) from Proteintech Group Inc. The membranes were scanned for statistical analysis by enhanced chemiluminescence (ECL) using a gel image processing system (Tanon, Shanghai) and the density of the bands was analyzed using Image J ( Wayne Rasband National Institutes of Health, USA).

### Animal experiments

The siRNA used *in vivo* was synthesized by Genepharma (Suzhou, China) and dissolved in PBS buffer. The dose of siRNA in nude mice was 0.5mg/kg.

10 BALB/c male nude mice aged 4-5 weeks (male, 18-22 g) were purchased from Beijing Vital River Laboratory Animal Technology and housed under a 12/12 hour light/dark cycle in an air-conditioned room at 22 ± 2°C with free food and water. All animal experiments were undertaken in accordance with the National Institute of Health Guide for the Care and Use of Laboratory Animals, with the approval of the Scientific Investigation Board of the College of Basic Medicine, Jilin University. The nude mice were randomly divided into two groups. Each of them was received 100 µL subcutaneous injection containing 5×10^5^ HCT15 cells. When the tumor size reached to 3-5 mm, siSSRP1 or NC (isodose PBS) was inoculated into the xenograft tumor by multi-point injection three times a week. Tumor size was measured every 3 days with a Vernier caliper and tumor volume was calculated with the following formula: V = (length) × (width) 2/2. After 34 days, mice were sacrificed by excessive intraperitoneal injection of barbiturates (Pentobarbital; 150 mg/kg; Spofa, Prague) followed by cervical dislocation. Tumor tissues were resected to be frozen at -80 °C for protein assay or fixed with 4% paraformaldehyde for hematoxylin-eosin (HE) staining and immunofluorescent staining.

### TUNEL assay

Tissue sections were treated by using One Step TUNEL Apoptosis Assay Kit (Beyotime Biotechnology Inc., Nantong, China) principally according to the instructions. TUNEL specimens were observed under the BX53 fluorescence microscope (Olympus, Japan) with a laser excitation at 488 nm to detect the FITC-labeled TUNEL-positive cells.

### Hematoxylin and eosin (H&E) staining and Immunohistochemistry

Tissues were fixed with 4% paraformaldehyde solution for at least 4 h at room temperature, followed by dehydration, dipping in wax, paraffin embedding and cut into sections. Then, these sections were treated with HE staining. For Immunohistochemistry, sections were incubated with serum or BSA for 30 min at room temperature, and then were dipped in diluted primary antibody for 2 h and then incubated with secondary antibody. The antibodies used in this research: primary antibodies: Proteintech (Wuhan, China): anti-SSRP1 (1:200; 15696-1-AP), anti-BCL2 (1:200; 26593-1-AP), anti-BAX (1:200; 50599-2-Ig), anti-MMP2 (1:200; 10373-2-AP), anti-MMP9 (1:200; 10375-2-AP) and PCNA (1:50; SC-56) from Santa Cruz Biotechnology; secondary antibody: goat anti-Rabbit IgG (H+L) (1:200; SA0000I-2) from Proteintech Group Inc. The sample was observed under BX53 fluorescence microscope (Olympus, Japan). Cells stained brown were positive cells.

### Statistical analysis

All analyses were performed using Microsoft Excel or Prism GraphPad 6.00. Data analyses were performed from at least three independent experimental groups. Comparison of the two sets of data was performed using the unpaired Student's t-test. To compare more than two sets, one-way analysis of variance analysis (ANOVA) with a Newman-Keuls multiple comparison test was conducted. For all experiments with error bars, the standard deviation was calculated to indicate the variation within each experiment. Values represent mean ± SEM. Differences were considered to be significant at *p < 0.05, **p < 0.01, vs. NC group.

## Results

### *SSRP1* expression was upregulated in both human colorectal cancer tissues and cells

**Figure 1:**
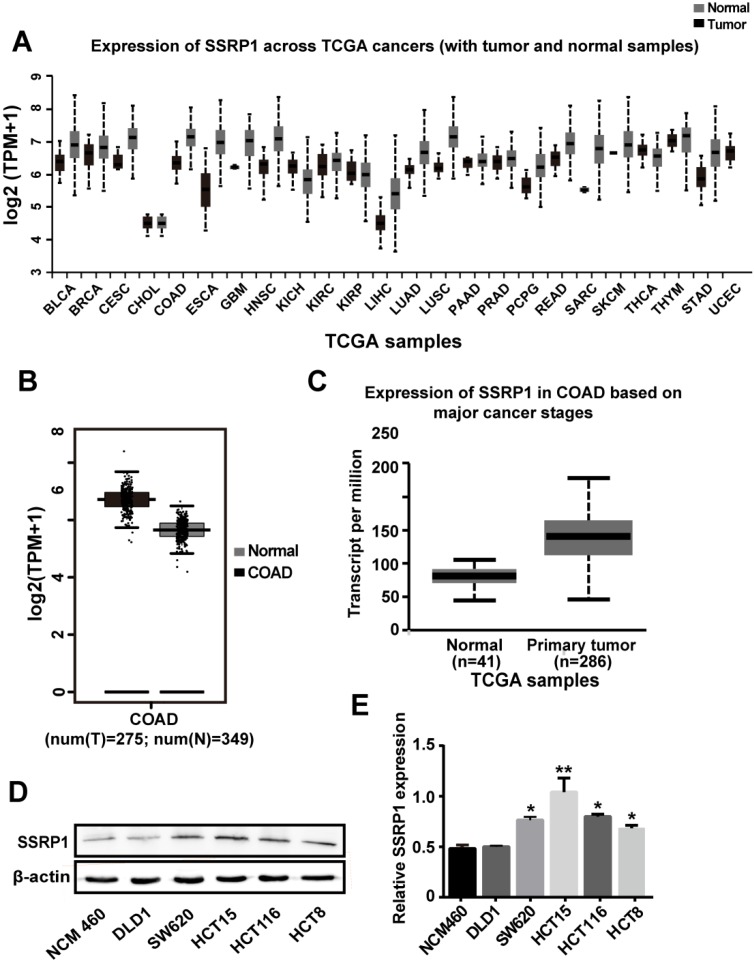
** SSRP1 expression is upregulated in both human colorectal cancer tissues and cells. (A)** UALCAN database displays SSRP1 is in a high expression status in multiple tumor types. **(B)** GEPIA database shows the expression of SSRP1is up regulated conspicuously in colorectal cancer tissues (n=275) versus normal tissues (n=349). (P< 0.01) **(C)** UALCAN database shows an obvious higher expression of SSRP1 in colorectal cancer patients (n=286) than normal patients (n=41) from TCGA. (*P*< 0.01) **(D, E)** Protein levels of SSRP1 in normal gastric cell line (NCM460) with a panel of colorectal cancer cell lines (DLD1, SW620, HCT15, HCT116, and HCT8).

Analysis of the *SSRP1* expression in human tumor tissues from the Firehose Broad GDAC database (https://gdac.broadinstitute.org/#) showed that *SSRP1* is upregulated in multiple tumor tissue types (Fig. 1A). Data from the Gene Expression Profiling Interactive Analysis (GEPIA; http://gepia.cancer-pku.cn/) also showed that the mean expression level of *SSRP1* in colorectal adenocarcinoma tissues was upregulated compared with the corresponding normal tissues (*P* < 0.01) (Fig. 1B). Results from the UALCAN database (http://ualcan.path.uab.edu/) also showed a higher level of *SSRP1* in patients with colorectal adenocarcinoma cancer than in normal patients (*P* < 0.001) (Fig. 1C). Furthermore, we compared a normal colorectal cell line (NCM460) with a panel of colorectal cancer cell lines (DLD1, SW620, HCT15, HCT116, and HCT8) for *SSRP1* expression and found that *SSRP1* was increased in the colorectal cancer cell lines (Figs. 1D, E). Based on the statistical data, we chose the HCT15 and SW620 cell lines for subsequent experiments.

### *SSRP1* inhibition repressed colorectal cancer cell proliferation

**Figure 2:**
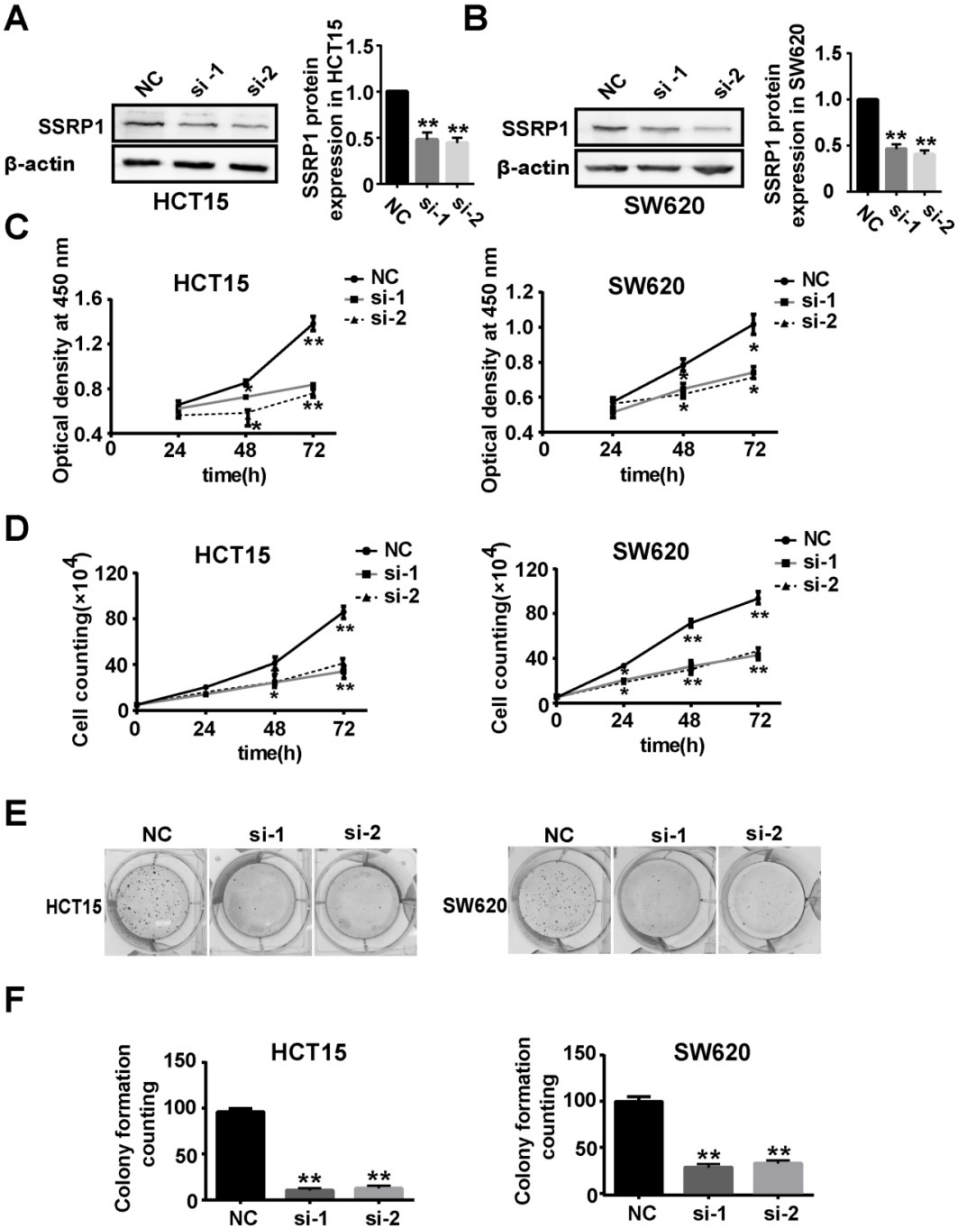
** SSRP1 inhibition represses proliferation of colorectal cancer cells. (A, B)** Protein levels of SSRP1 in HCT15 and SW620 cells at 48 h post siRNA transfection and densitometric quantification of proteins normalized to β-actin. **(C)** CCK8 assay after siRNA transfection for 48 h at OD 450 nm in HCT15 and SW620 cells. **(D)** The growth curve of HCT15 and SW620 cells after siRNA transfection. **(E, F)** Colony formation ability of HCT15 and SW620 cells after SSRP1 silence by transfecting siRNA. *P< 0.05, **P< 0.01 vs NC group.

To explore the function of SSRP1 in the development of colorectal cancer, we used RNAi technology to silence the expression of *SSRP1* in colorectal cancer cells to observe whether *SSRP1* modulation affected colorectal cancer cells. After siRNA transfection for 48 h, SSRP1 protein levels in HCT15 and SW620 cells were downregulated, as assessed by western blotting (Fig. 2A, B). Supplementary Fig. S1A and B provide the data on the *SSRP1* silencing in HCT116 cells.

We next examined the effects of SSRP1 on HCT15 and SW620 cell proliferation. Compared with cells transfected with the negative control (NC), the HCT15, SW620, and HCT116 cell viabilities at OD 450 nm decreased by different degrees after siRNA transfection for 24, 48 and 72 h (Fig. 2C, Supplementary Fig. S1C). Cell proliferation was also assessed using cell counting and colony formation assays. As expected, the cell number and colony formation ability of the HCT15 and SW620 cells decreased significantly after *SSRP1* siRNA transfection (Fig. 2D, E, F). HCT116 cells treated with siRNA also exhibited weakened colony formation ability (Supplementary Fig. S1D, E, F). These data revealed that *SSRP1* repressed the proliferation of colorectal cancer cells.

### SSRP1 affected proliferation and apoptosis of colorectal cancer cells by inhibiting the AKT signaling pathway

**Figure 3:**
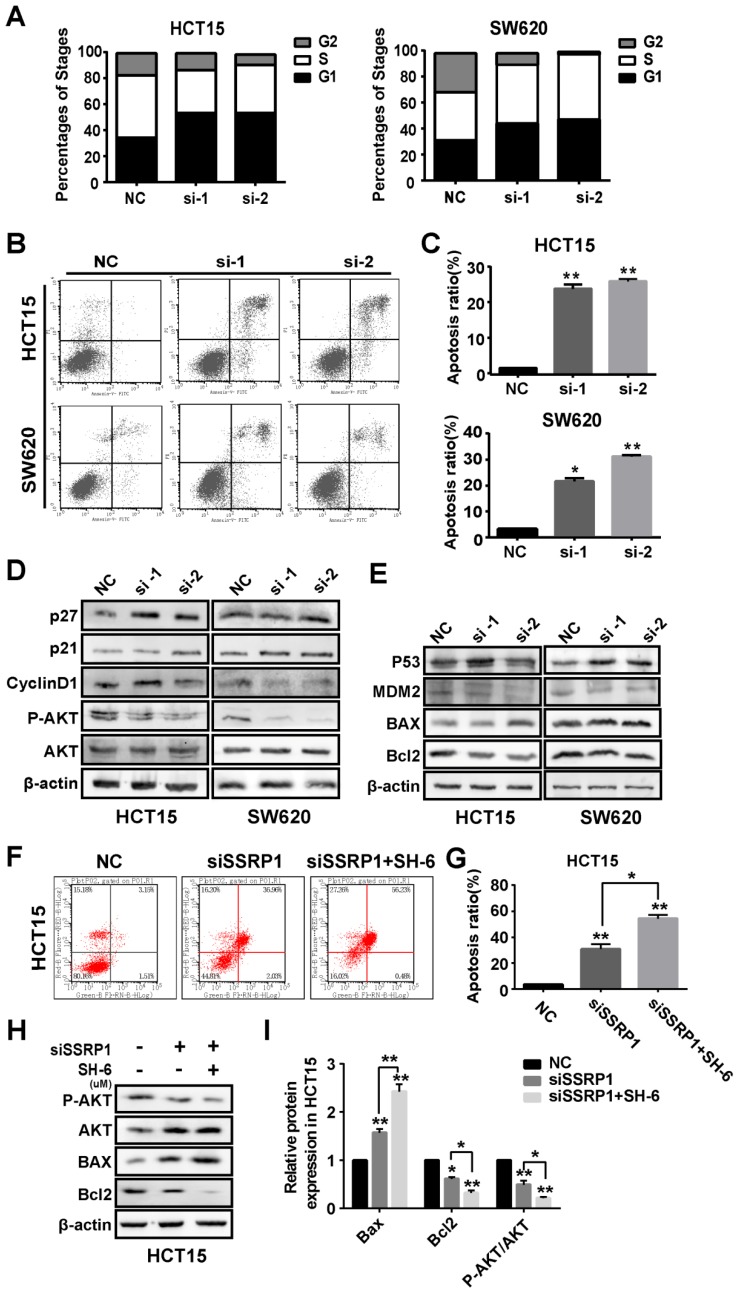
** SSRP1 exerts proliferation and apoptosis of colorectal cancer cells by activating the AKT pathway. (A)** Graphs with quantitative data for the flow cytometric cell cycle distribution assay in HCT15 and SW620 cells. **(B, C)** FITC Annexin V/PI staining indicated increased apoptosis in HCT15 and SW620 cells with SSRP1 inhibiting. **(D, E)** Western blot displayed reduced P-AKT, CyclinD1, BCL-2 and MDM2, accompanying increased p27, p21, p53 and BAX in transfected HCT15 and SW620 cells. **(F, G)** Apoptosis ratio in HCT15 cells with SSRP1 or AKT inhibitors through flow cytometry. **(H, I)** The protein level of P-AKT, AKT, BAX and BCL-2 in HCT15 cells with SSRP1 or AKT inhibitors. *P< 0.05, **P< 0.01 vs NC group.

Based on the observed effects of SSRP1 on the proliferation of colorectal cancer cells, we examined the cell cycle and apoptosis of colorectal cancer cells by flow cytometry to further explore SSRP1 regulation of colorectal cancer cell proliferation. HCT15 and SE620 cells treated with siRNA for 48 h underwent significant G1 phase arrest compared with cells treated with NC (Fig. 3A, Supplementary Fig. S2A). The percentage of apoptotic cells in the siRNA-treated group was significantly increased (Fig. 3B, C).

The AKT pathway is a classic signaling pathway involved in proliferation and apoptosis. To determine how SSRP1 regulates the cell cycle and apoptosis, we used western blotting to detect alterations in critical proteins in the AKT signaling pathway. Figure 3D shows that *SSRP1* knockdown reduced AKT phosphorylation (P-AKT) and altered the cell cycle regulators, p21, p27 and cyclin D1 (Fig. 3D, Supplementary Fig. S2B). Furthermore, we found that downstream BAX expression was increased and BCL2 expression was decreased (Fig. 3E, Supplementary Fig. S2C). Significant increase of apoptotic rate and apoptosis-related protein in HCT15 cells treated with SSRP1 silence and SH-6 using western blot and flow cytometry (Figure 3F-I), illustrating the inhibition of AKT signaling pathway indeed could induce apoptosis in colorectal cancer cells.

Therefore, *SSRP1* silencing may activate the AKT signaling pathway to block proliferation and promote apoptosis.

### Downregulation of *SSRP1* inhibited migration and invasion of colorectal cancer cells

**Figure 4:**
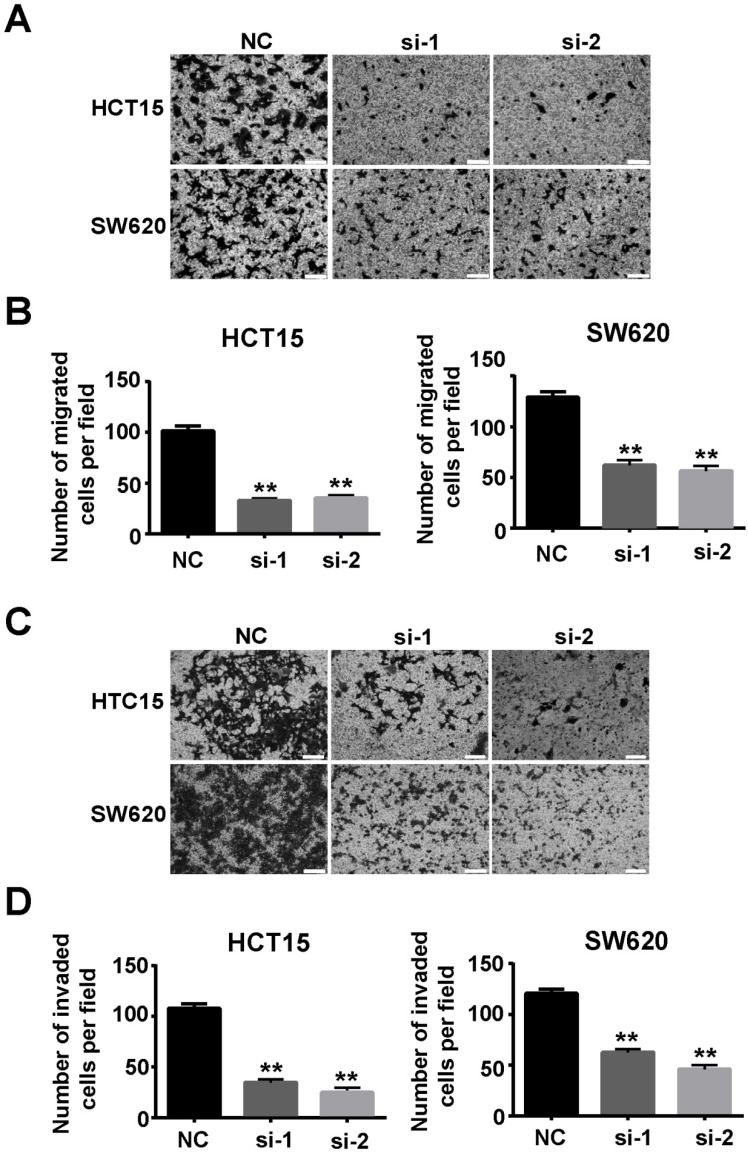
** Downregulation of SSRP1 inhibits migration and invasion of colorectal cancer cells.** Migration **(A, B)** and invasion **(C, D)** ability of HCT15 and SW620 cells through transwell assay after SSRP1 downregulation. *P<0.05, **P<0.01 vs NC group.

Our study also verified that *SSRP1* affected migration and invasion of colorectal cancer cells. Transwell assays showed that downregulation of *SSRP1* using siRNA impaired the migration (Figs. 4A, B) and invasion (Figs. 4C, D) of both HCT15 and SW620 cells.

### *SSRP1* inhibition blocked colorectal cancer growth *in vivo*

**Figure 5:**
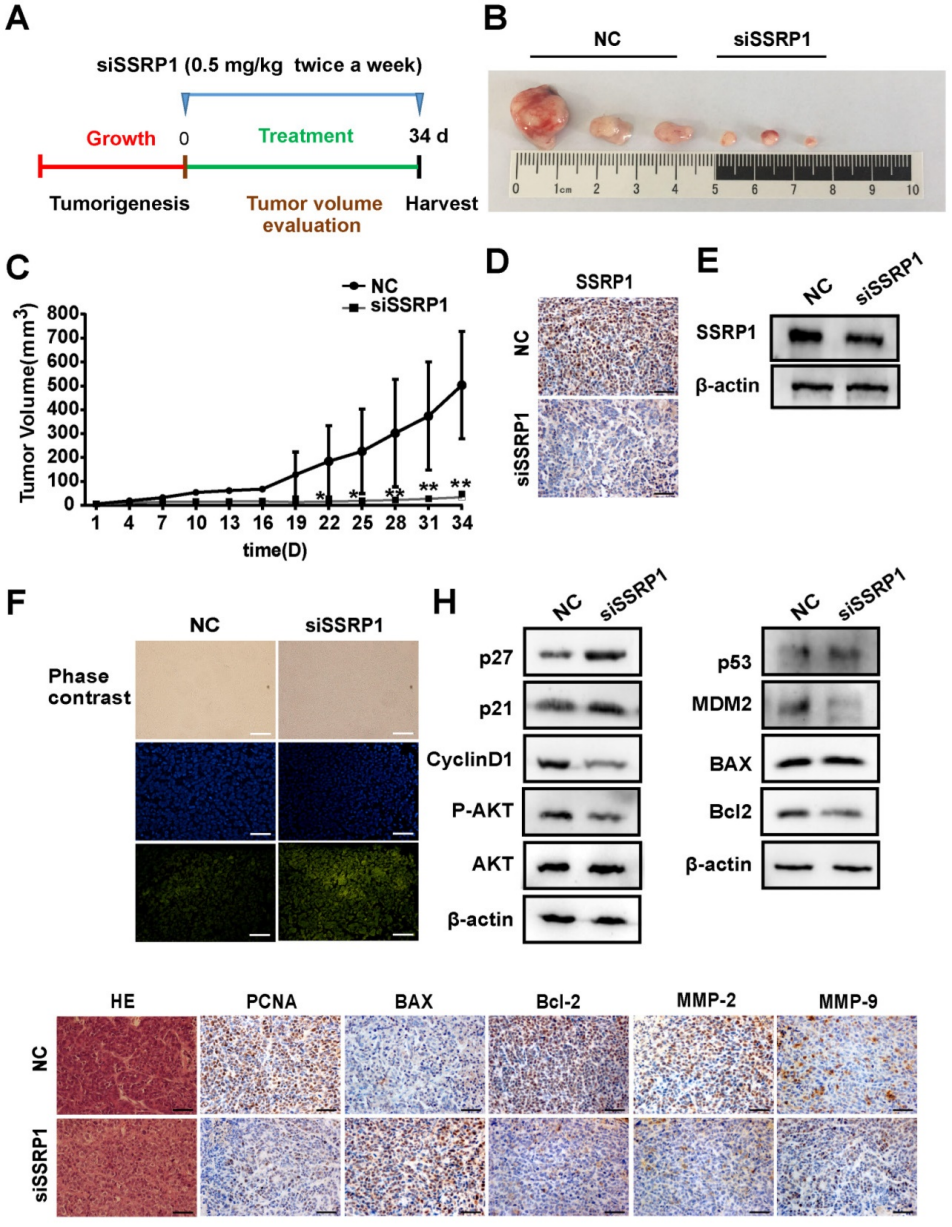
** SSRP1 inhibition blocks the growth of colorectal cancer *in vivo*. (A)** Treatment for transplanted xenogeneic models using HCT15 cells. **(B)** Image of the tumor tissues of NC group and siSSRP1 group. **(C)** Tumor growth curve of NC group and siSSRP1 group for 34 d treatment. **(D)** Immunofluorescent staining showing that SSRP1 expression was reduced in siSSRP1 group, compared with NC group. **(E)** Validation of SSPR1 protein levels through western blot. **(F)** TUNEL assay of tumor tissue. **(G)** Immunofluorescent staining of PCNA, BAX, Bcl2, MMP-2 and MMP-9. **(H)** AKT, P-AKT, p27, p21, CyllinD1, p53, MDM2, BAX, and Bcl2 protein levels were determined by western blot analysis. *P<0.05, **P<0.01 vs NC group.

Since *SSRP1* knockdown altered the proliferation and apoptosis of colorectal cancer *in vitro* as well as the migration and invasion of colorectal cancer cells, we attempted to determine whether the same phenomenon would occur *in vivo*. We used the HCT15 cell line to construct a colorectal cancer xenograft model and randomly divided nude mice into the si*SSRP1* and NC groups. When the tumor size reached 3-5 mm, either si*SSRP1* (0.5 mg/kg) or NC was inoculated into xenograft tumors by multipoint injection three times per week (Fig. 5A). The si*SSRP1* group exhibited a significantly smaller tumor volume and growth rate (Fig. 5B, C); however, no significant differences occurred in body weight between the two groups (Supplementary Fig. S3A). We also verified the expression of SSRP1 by western blotting and immunohistochemistry. The expression of SSRP1 in the si*SSRP1* group was markedly downregulated (Fig. 5D, E). TUNEL assay was performed to detect the apoptotic cells, which demonstrated that the si*SSRP1* group had strong green fluorescence intensity at the same exposure time (Fig. 5F). This indicated that that *SSPR1* silencing induced apoptosis in the colorectal cancer cells *in vivo*. Moreover, the results of immunohistochemistry also confirmed that downregulation of *SSRP1* could undermine the expression of PCNA, BCL-2, MMP-2 and MMP-9, and at the same time, it also induced the an increase in the expression of BAX (Fig. 5G). Consistent with the *in vitro* results,* SSRP1* knockdown *in vivo* activated the AKT signaling pathway and altered the expression of downstream proteins related to the cell cycle and apoptosis. Collectively, these data showed that downregulation of *SSRP1* inhibited colorectal cancer progression *in vivo*.

## Discussion

The histone chaperone protein, FACT, is composed of SSRP1 and SUPT16H (suppressor of Ty 16 homolog) and assists in unraveling DNA molecules from histone octamers, reduces the compactness of nucleosomes, and promotes extension during transcription 9, 10. It is also closely related to cell proliferation and apoptosis 11. Current research on *SSRP1* indicates that it is upregulated in various tumor types^5^, ^12^. Moreover, changes in *SSRP1* expression affect tumor characteristics; for example, curaxins, inhibitory compounds with specific activity against FACT, induce apoptosis in tumor cells by activating p53 and inhibiting NF-κB 13. Downregulation of *SSRP1* expression by siRNA interference also results in the proliferation of U87 and U251 glioma cells through the MAPK pathway 8. In addition, lower FACT expression has been reported in more differentiated cells compared with stem cells, progenitor cells, and less differentiated cells, and expression levels changed after experimentally inducing differentiation 14. Our findings and those of previous studies suggest that *SSRP1* may be a target gene for cancer therapy, but further work is required to determine the potential function of SSRP1 in colorectal cancer progression.

Colorectal cancer can be divided into three histological types: adenocarcinoma, mucinous carcinoma, and undifferentiated carcinoma. Colorectal adenocarcinoma accounts for approximately three-quarters of colorectal cancer cases. Our analysis of data from TCGA confirmed that *SSRP1* is upregulated in many human tumor tissues, including colorectal adenocarcinoma, along with multiple colorectal cancer cell lines. We also found that, compared with normal patients, colorectal adenocarcinoma patients showed significantly increased SSRP1 expression levels, as demonstrated by histochemical staining results from the Human Protein Atlas database. This indicated that *SSRP1* may act as an oncogene in the development of colorectal cancer. To verify this, we downregulated the *SSRP1* expression using siRNA and found that SSRP1 inhibition significantly inhibited proliferation and metastasis and promoted apoptosis of colorectal cancer *in vivo* and *in vitro.* However, the small number of tumor tissues was still a shortcoming in our research process.

The PI3K-AKT signaling pathway is a classic signaling pathway associated with cell survival and apoptosis, and the serine/threonine kinase, AKT (also known as protein kinase B), which comprises a group of three isoforms (AKT1, AKT2, and AKT3) in mammals, is a critical propagator of PI3K signaling 15,16.

Activated AKT phosphorylates many substrates controlling almost every aspect of various physiological and pathological cellular functions, including cell survival, growth, metabolism, tumorigenesis, and metastasis 17-19. We thus investigated whether the AKT signaling pathway participated in the changes in proliferation and apoptosis in colorectal cancer after SSRP1 intervention. Our results revealed that *SSRP1* silencing activated the AKT signaling pathway to regulate proliferation, metastasis, and apoptosis in colorectal cancer.

However, further research to clarify the molecular mechanism of the interaction between *SSRP1* and AKT pathway. In summary, the present study confirmed that *SSRP1* influences the proliferation and apoptosis of colorectal cancer cells via the AKT pathway.

## Supplementary Material

Supplementary figures.

## Abbreviations

SSRP1: structure-specific recognition protein 1
TCGA: the Cancer Genome Atlas
