# Supplementary material for: SSRP1 influences colorectal cancer cell growth and apoptosis via the AKT pathway
Source: Int J Med Sci. 2019 Oct 21;16(12):1573–82. doi: 10.7150/ijms.38439 (PMC6909804; doi:10.7150/ijms.38439)
Supplement: Supplementary file 1 — Supplementary figures. [file ijmsv16p1573s1.pdf]

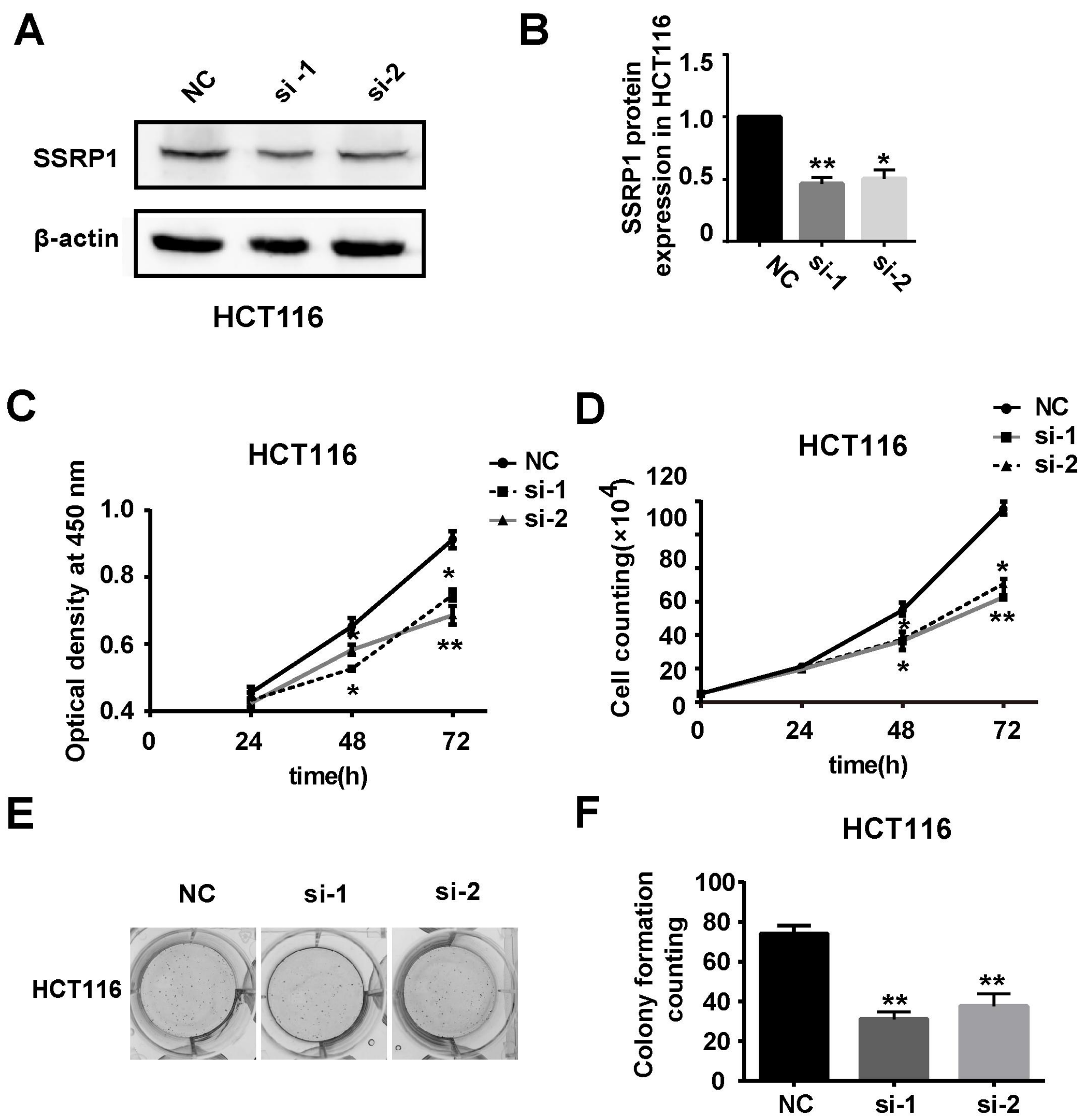

Supplementary Fig. S1. SSRP1 inhibition represses proliferation of colorectal cancer cells.

(A, B) Protein levels of SSRP1 in HCT116 cells at 48 h post siRNA transfection and densitometric quantification of proteins normalized to β-actin. (C) CCK8 assay after siRNA transfection for 48 h at OD 450nm in HCT116 cells. (D) The growth curve of HCT15 after siRNA transfection. (E, F) Colony formation ability of HCT116 cells after SSRP1 silence. * *P*<0.05, ** *P*<0.01 vs NC group.

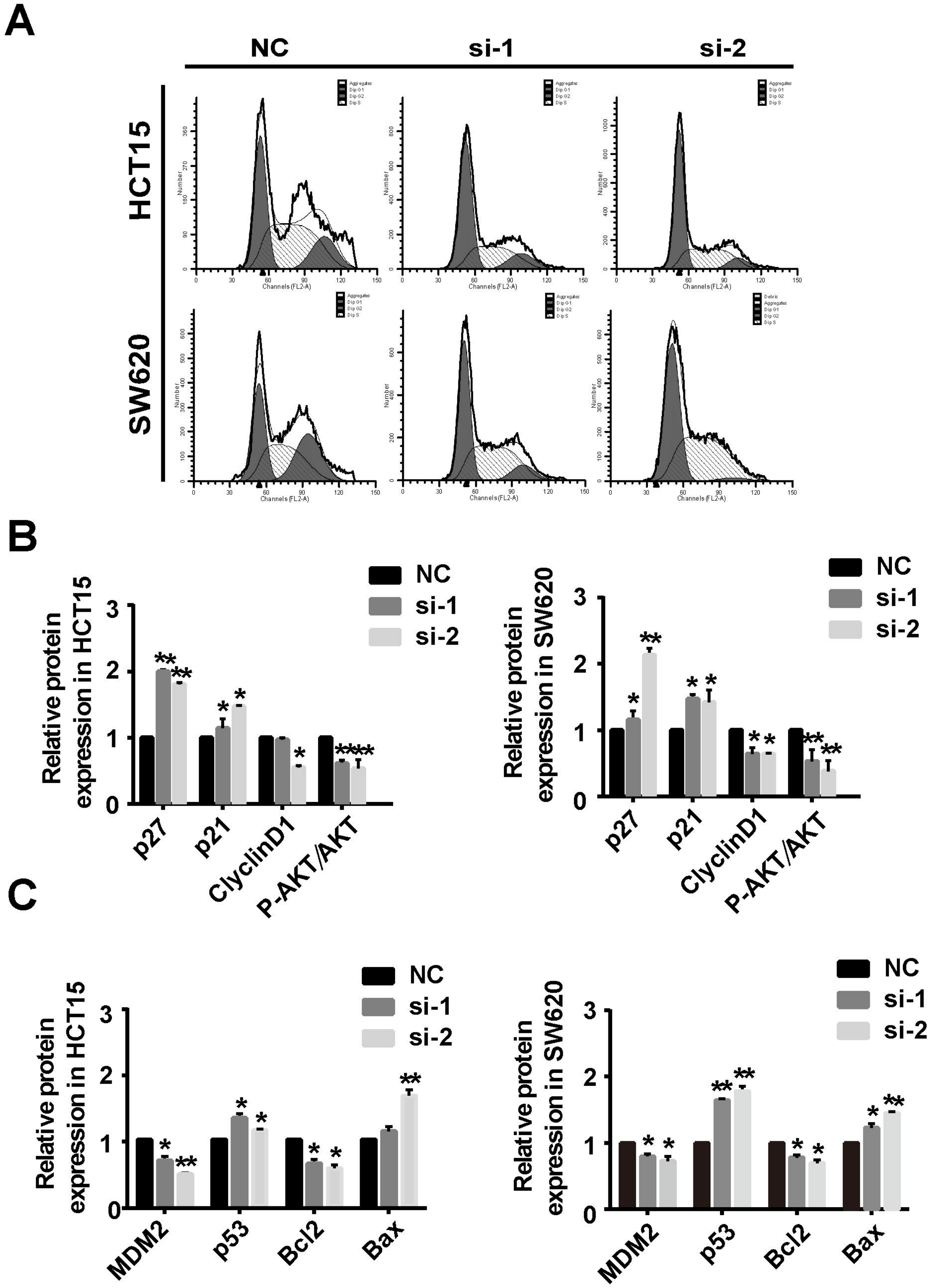

Supplementary Fig. S2. SSRP1 exerts proliferation and apoptosis of colorectal cancer cells by activating the AKT pathway.

(A) Flow cytometric cell cycle distribution assay in HCT15 and SW620 cells. (B, C) Graphs with densitometric analysis of the protein bands in HCT15 and SW620 cells. * $P<0.05$, ** $P<0.01$ vs NC group.

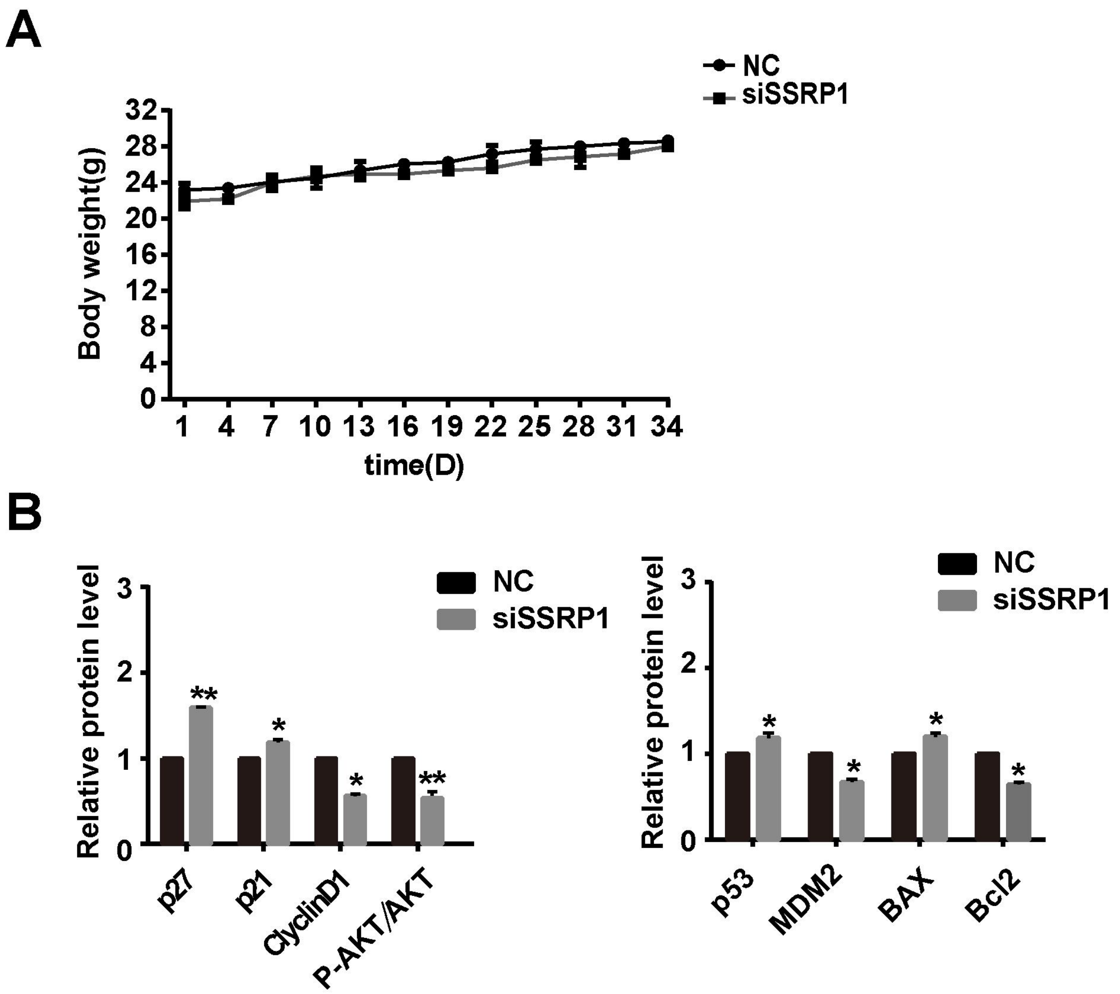

Supplementary Fig. S3. SSRP1 inhibition blocks the growth of colorectal cancer *in vivo*.

(A) Statistical results of changes in body weight within 34 days of mice. (B) Densitometric analysis of the protein from tissues. * P<0.05, ** P<0.01 vs NC group.
